# The cortisol awakening response in very preterm born adults compared to term born adults

**DOI:** 10.1111/jne.70000

**Published:** 2025-02-10

**Authors:** Bilge Albayrak, Giorgi Batsikadze, Lara Jablonski, Ursula Felderhoff‐Müser, Tina Hörbelt‐Grünheidt, Anna Lena Friedel, Raphael Hirtz, Katharina Heuser‐Spura, Monia V. Dewan

**Affiliations:** ^1^ Department of Pediatrics I and Center for Translational Neuro‐ and Behavioral Sciences (C‐TBNS) University Hospital Essen, University of Duisburg‐Essen Essen Germany; ^2^ Department of Neurology and C‐TBNS University Hospital Essen, University of Duisburg‐Essen Essen Germany; ^3^ Institute of Medical Psychology and Behavioral Immunobiology and C‐TBNS University Hospital Essen, University of Duisburg‐Essen Essen Germany; ^4^ Division of Pediatric Endocrinology and Diabetology, Department of Pediatrics II University Hospital Essen, University of Duisburg Essen Essen Germany; ^5^ Center for Child and Adolescent Medicine Helios University Hospital Wuppertal, Witten/Herdecke University Wuppertal Germany

**Keywords:** anxiety, cortisol awakening response, development, stress response, very preterm infants

## Abstract

Very preterm infants are at higher risk of long‐term neurodevelopmental and psychiatric impairments, including anxiety. Prematurity is also linked to altered programming of the hypothalamus–pituitary–adrenal (HPA) axis, associated with stress‐related diseases. The cortisol awakening response (CAR), marked by a rapid cortisol increase after waking, is a biomarker of HPA reactivity and is linked to psychiatric disorders. This study aimed to evaluate for the first time the CAR in adults born very preterm and to explore its association with anxiety and stress. Twenty‐five young adults born very preterm and 24 age‐ and sex‐matched term‐born controls collected saliva samples on two consecutive mornings at 0, 30, 45, and 60 min after awakening. Anxiety was measured using the State–Trait Anxiety Inventory, and stress was assessed with the Perceived Stress Scale. The CAR was analyzed using the sample at 0 min (S1), total cortisol output (AUCg), and actual CAR (AUCi). There were no significant differences in AUCi. The preterm group exhibited lower S1 levels and a reduced AUCg. Preterm‐born participants reported higher trait anxiety and stress, though no consistent link with the CAR was identified. Findings suggest long‐term neuroendocrine changes in adults born very preterm, warranting further research.

**Clinical Trial Registration:** Duetsche Register Klinischer Studien (DRKS): 00020235.

## 1 INTRODUCTION

Very preterm infants (VPT) (infants born before 32 weeks' gestation) are at increased risk of neurodevelopmental impairments as birth coincides with a vulnerable period of brain development.^1^, ^2^ Neurodevelopmental impairments include mental disorders, from which preterm‐born children and adults are two‐ to fourfold more affected compared to term‐born children and adults.^3^, ^4^ Besides attention deficit hyperactivity disorders and autism spectrum disorders, anxiety is most prevalent among preterm‐born children, which may persist into adulthood.^3^, ^5^ Anxiety disorders are associated with altered functioning of the hypothalamus–pituitary–adrenal (HPA) axis.^6^ Preterm infants show signs of HPA axis alterations in the short and in long term.^7^ Shortly after birth and in the first week of life, preterm‐born infants might suffer from relative adrenal insufficiency (RAI) with symptoms such as hypoglycemia or hypotension.^7^ RAI describes insufficient glucocorticoid secretion in response to stress and illness^8^ due to immaturity of the HPA axis.^7^ Although the long‐term alterations of the HPA axis in preterm‐born children and adults are not fully understood yet, there is evidence of increased basal cortisol levels and impaired cortisol reactivity to stress.^7^, ^9^, ^10^ The altered programming of the HPA axis might be a consequence of pre‐ and postnatal stress exposure, for example, repeated painful procedures during treatment on the neonatal intensive care unit (NICU).^11^, ^12^

A psychoneuroendocrine biomarker for HPA axis reactivity is the cortisol awakening response (CAR). The CAR describes the physiological increase in cortisol secretion during the first 30–45 min after awakening in the morning (Staider et al., 2016). The CAR superimposes the circadian rhythm of the diurnal cortisol secretion. It is important to note that the CAR is considered a distinct entity from the cortisol levels during the rest of the day.^13^ Alterations of the CAR, for example, insufficiently low or excessively high cortisol morning levels upon awakening are associated with stress related disorders such as cardiovascular diseases, major depression, and anxiety.^14^ While studies on the CAR in children born preterm are inconclusive with some studies reporting increased^15^ and others reporting decreased CAR,^16^, ^17^ it has not been investigated in young adults yet.

Recently, our group showed increased anxiety levels in very preterm born young adults compared to age‐ and sex‐matched term born adults.^18^ Considering these findings, the study had two main objectives. First, we aimed to investigate the effect of prematurity on the CAR. Second, we investigated its association with anxiety and stress within the same cohort. We hypothesized that the CAR differs between very preterm and term‐born adults and that it is associated with increased stress and anxiety levels in the preterm‐born group.

## 2 METHODS

### 2.1 Participants

The original cohort comprised a total of 37 preterm‐born participants (19 women/18 men, mean age 20.2 ± 2.8 years) and 31 controls (16 women/15 men, mean age 22.4 ± 2.3 years), who were recruited to perform a differential fear‐conditioning paradigm, an associative learning process, as previously published.^18^

Inclusion criteria were as follows: (i) very preterm birth (23–32 weeks' gestation) or term birth (≥37 weeks' gestation), and (ii) age‐appropriate development at the time of the testing without special needs/education. Exclusion criteria were: (i) intra/(peri‐) ventricular hemorrhage ≥ III or periventricular leukomalacia based on brain MRI or ultrasound acquired at the time of term equivalent age, (ii) focal neurological disorders, (iii) chronical diseases with and without regular medication. For clinical description of the included participants see Table 1. Participants were then additionally invited to participate in the study on CAR. Upon agreement to participate, they received, either in person or via mail, a box containing instructions, eight commercial saliva collection devices (Salivette® Cortisol; Sarstedt, Nuembrecht, Germany), and questionnaires, including the State–Trait Anxiety Inventory (STAI), Perceived Stress Scale (PSS), and Stanford Sleepiness Scale (SSS). Additionally, each participant was provided with an instructional video.

**TABLE 1:** Group characteristics of very preterm adults and controls.

	Very preterm (*n* = 25)	Term (*n* = 24)	*p* ^a^
Clinical characteristics			
Gestational age, weeks [range]	29 + 1 [26 + 2–32.0]	39 + 5.7 [37.0–42.0]	*t*_47_ = −23.22, *p* < .**001**
Birth weight, grams [range]	1240.4 [850–1750]	3648.3 [3020–5360]	*t*_46_ = −18.04, *p* < .**001**
Female, *n* (%)	14 (56%)	14 (58.3%)	*Χ* ^2^ = 0.03, *p* = .869
IVH < grad III, *n* (%)	4 (16%)	0 (0%)	*p* = .101
Follow‐up characteristics			
Age at assessment, years [range]	20.05 [17.8–27]	22.03 [18–25]	*t*_47_ = −3.00 *p* = .**002**
Education, high^b^, *n*	19	24	*p* = .**022**
Parental education, high^b^, *n*	22	24	*p* = .235
Health status^c^	4.95 [3.73–6.37]	5.63 [4.33–6.89]	*t*_47_ = −3.23, *p* = **−.001**
IQ	100.4 [88–123]	108.5 [89–122]	*t*_47_ = −3.29, *p* < .**001**
Any therapy^d^, *n* (%)	15 (60%)	0 (0%)	*p* < .**001**
Any psychiatric/social–emotional disorders^e^, *n* (%)	6 (24%)	1 (3.2%)	*Χ* ^2^ = 3.93, *p* = .**047**
Phobias/anxiety/depression, *n* (%)	1 (4%)	0 (0%)	*p* = 1.0
ADS/ADHS, *n* (%)	5 (20%)	0 (0%)	*p* = .0502
Developmental disorders^f^, *n* (%)	4 (16%)	0 (0%)	*p* = .11
SSS Day 1 [range]	2.72 [1–5]	2.63 [1–4]	*U* = 294.5, *p* = .920
SSS Day 2 [range]	3.32 [1–6]	2.88 [1–6]	*U* = 233, *p* = .184
BMI	21.9 [18–28.4]	22.9 [18.8–27.5]	*t*_47_ = −1.78, *p* = .122
Use of contraceptives	11 (78.6%)	13 (92.3%)	*Χ* ^2^ = 1.17, *p* = .280

*Note*: Data are presented as mean (± standard deviation) if not indicated otherwise. Bold indicates significant value (*p* < .05).

^a^ *t*‐test or Mann–Whitney *U*‐test and chi‐square results or Fischer's exact test for continuous and categorical data, respectively.

^b^ >10 years school.

^c^ Assessment of health status based on the *Life Satisfaction Questionnaire (Fragebogen zur Lebenszufriedenheit, FLZ)*.

^d^ Having any therapies, including speech therapy, physical therapy, or occupational therapy.

^e^ Psychiatric disorders including attention‐deficit‐(hyperactivity)‐disorder, emotional disorder. SGA—small for gestational age (birth weight < 10th percentile), IVH—intraventricular hemorrhage, BPD—bronchopulmonary dysplasia, ROP—retinopathy of prematurity, IQ—intelligence quotient based on the *Wechsler Adult Intelligence Scale—Third Edition*.

^f^ Developmental disorders concerning language, gross‐ or fine motor functions which needed therapy in the past (speech therapy, physical therapy or occupational therapy).

The final cohort included 25 preterm‐born participants (14 women/11 men, mean age 20.1 ± 2.6 years) and 24 controls (14 women/10 men, mean age 22 ± 1.9 years), who agreed to participate in this secondary study.

The Ethics committee of the University of Duisburg‐Essen approved the study (19‐8890‐BO). For the current study an amendment was approved. The study was performed following the principles laid down in the Declaration of Helsinki. All participants gave written informed consent.

### 2.2 Saliva samples

Saliva samples for CAR analysis were obtained following the consensus guidelines of the International Society of Psychoneuroendocrinology.^19^ Participants were instructed to choose two regular consecutive days to retrieve cortisol samples on certain points of time after awaking (0, 30, 45, and 60 min). Saliva was collected with a commercial collection device (Salivette® Cortisol; Sarstedt, Nuembrecht, Germany). Participants were asked to choose days without any special occasion and to wake up until 8 a.m. the latest. As soon as they woke up participants were supposed to retrieve the first cortisol sample (0 min) then to stand up, lighten the room and to wander around to wake up completely. They were asked not to eat, drink, smoke or brush teeth before the end of the sampling. To ensure that sampling was performed comparably hand‐outs with drafts were distributed and personally explained. A video tutorial explaining the hand‐outs and showing how to retrieve the samples was provided for home use. Furthermore, participants had to fill out a checklist including time of waking up, time of saliva sampling, checking for undesirable behavior (e.g., smoking, drinking), checking for illumination of the room and for direct storing the probes in the refrigerator. Samples had to be returned up to 2 weeks after collection. When sent back to the laboratory, saliva samples were stored at –80° after centrifugation until analysis. Salivary cortisol concentrations were analyzed by enzyme‐linked immunosorbent assay (Cortisol Saliva ELISA, IBL International, Hamburg, Germany) according to the manufacturer's instructions by trained stuff in a laboratory that regularly takes part in round‐robin tests for quality assurance and method validation. Cross‐reactivity of the anti‐cortisol antibody with other relevant steroids was 8.5% (11‐deoxycortisol), 2.6% (cortisone), 1.0% (corticosterone), and <0.1% (estrone, estradiol, estriol, progesterone, testosterone). Inter‐ and intra‐assay coefficients of variation were <10%.^20^

### 2.3 Questionnaires

Between the 0‐ and 30‐min sample, participants were instructed to fill out several questionnaires: the Perceived Stress Scale (PSS), the State–Trait Anxiety Inventory (STAI) and the Stanford Sleepiness Scale (SSS) on Day 1. On Day 2, only the State‐Anxiety part of the STAI and the SSS had to be filled out.

### 2.4 State–Trait Anxiety Inventory (STAI)

The STAI consists of a state and a trait anxiety scale, each containing 20 questions. These questions have to be answered on a four‐point Likert‐scale ranging from 1 “almost never” to 4 “almost always.” Total scores range from 20 to 80. Higher scores indicate higher levels of state/trait anxiety,^21^ whereby trait anxiety reflects a general and lasting tendency to react with anxiety, while state anxiety displays a momentary emotional state whose in intensity is variable over time and situation.

### 2.5 Perceived Stress Scale—10 (PSS‐10)

The PSS‐10 measures the extent to which situations in a person's life are perceived as stressful. Participants have to answer 10 questions about thoughts and feelings during the last month on a five‐point likert‐scale ranging from “never” to “very often.” The maximum score is 50, with higher total scores indicating a higher perceived stress level.^22^

### 2.6 Stanford Sleepiness Scale (SSS)

To check on subjective sleepiness, the SSS was used. The SSS consists of only one item with seven statements to quantify subjective sleepiness levels. The higher the score, the higher the level of sleepiness.^23^

### 2.7 Statistics

The normality of the data for salivary cortisol, AUCg, and AUCi was checked using the Shapiro–Wilk test, and all variables were found to be normally distributed (all *p* > .05). Statistical analysis was conducted using repeated measures ANOVA (RMANOVA) in SAS (SAS Studio 3.8, SAS Institute Inc., Cary, NC, USA). Concerning these analyses, AUCg or AUCi were the dependent variables as previously described.^20^ AUCg represents the total cortisol output over the time period measured in this study, while AUCi is an index of cortisol change during the observed period, reflecting the CAR.^24^ The within‐subject factor was Day (Day 1 or Day 2) and the between‐subject factor was Group (preterm‐born and term‐born adults). Post hoc comparisons utilized least‐squares means tests and were adjusted for multiple comparisons using the Bonferroni method. Post hoc tests were conducted when time or group factor or the interaction was significant.

A further important parameter is the first cortisol level upon awakening at 0 min (S1).^19^

Additional RMANOVAs were conducted using salivary cortisol as dependent variable, time (0, 30, 45, and 60 min) as the within‐subject and Group (preterm‐born and term‐born adults) as between subject factor, separately for each day. Correlations and comparative analyses were performed on physiological outcome measures. To investigate the relationship between AUCg and AUCi with state anxiety, trait anxiety, and perceived stress separately, Pearson correlations were calculated for all pairs of measures, separately for each day.

State anxiety, trait anxiety, and perceived stress scores (PSS) were compared between groups using the Mann–Whitney *U* test due to the ordinal nature of the data.

We considered sex and contraceptive use as important covariates in the RMANOVAs, given their potential influence.^19^ However, after adjusting for these factors, they were not statistically significant and did not have an impact on AUCg or AUCi, indicating no measurable effect on these specific outcomes within the context of this study (Table S1).

## 3 RESULTS

### 3.1 Participants' characteristics

From the original study group, 25/38 preterm born adults and 24/42 term born adults agreed to participate in the add on study. Clinical characteristics are depicted in Table 1. Sex distribution was similar between the two groups. The mean age was significantly lower in the preterm group with 20.5 years (range, 17.8–27) compared to 22.3 years (range, 18–25). The control group was significantly higher educated compared to the preterm group (*p* = .02). The mean IQ was higher in the control group (*p* = <.001). The prevalence of psychiatric and social–emotional disorders was significantly higher among preterm adults. Subjective sleepiness levels assessed with the SSS on Day 1 and Day 2 did not reveal any differences between the preterm and term group. Please see Table S2 for the missingness analysis.

### 3.2 Cortisol awakening response

**FIGURE 1:**
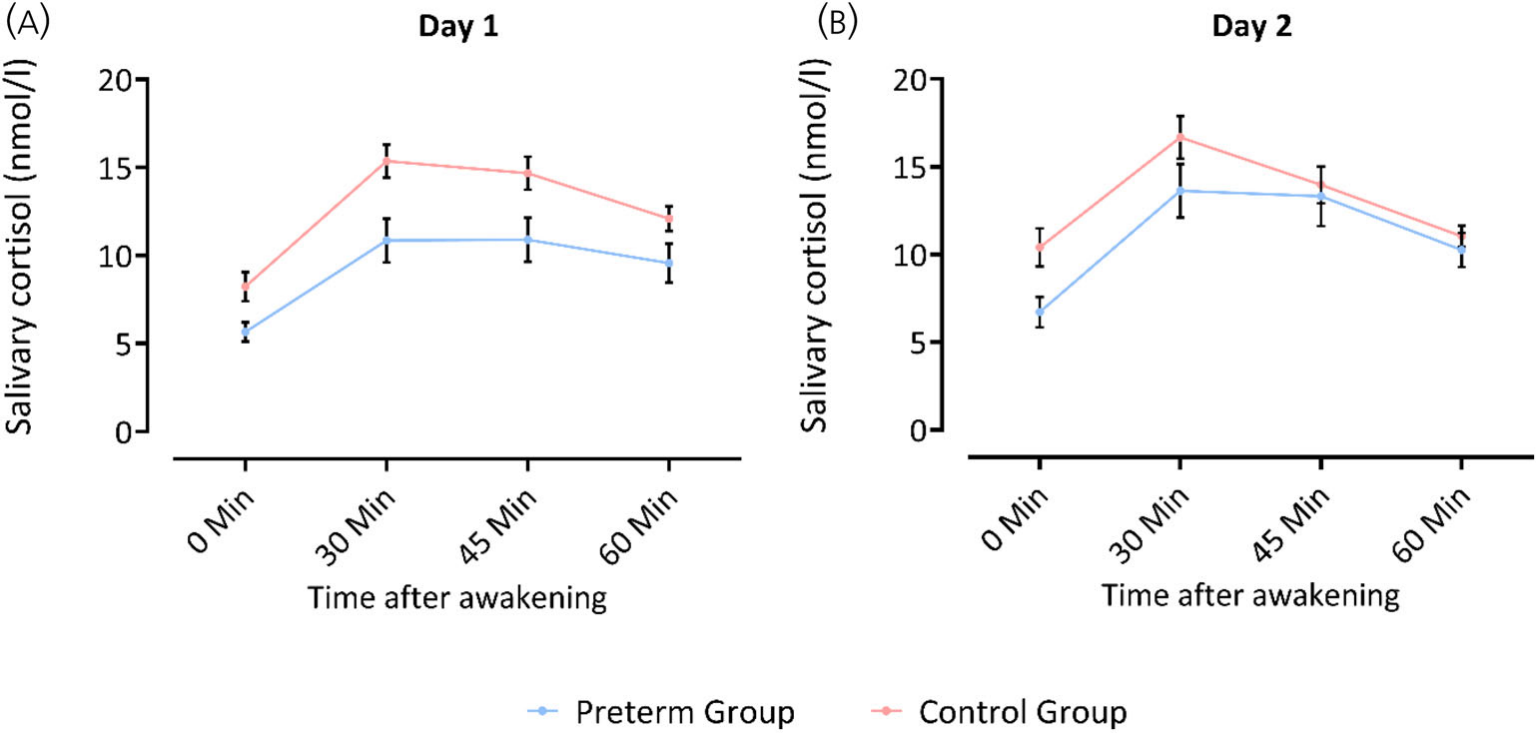
Course of salivary cortisol concentration for the preterm (blue) and control (red) group. The x‐axis presents the four time points (minutes after waking up); the y‐axis presents the salivary cortisol levels in nmol/L. (A) Day 1; (B) Day 2. *n* = 25 for the preterm and *n* = 24 for the control group for each time point.

Figure 1 summarizes preterm and term born adults' CAR at 0, 30, 45, and 60 min after awakening. For all participants, salivary cortisol increased after awakening as expected, irrespective of being born preterm or full‐term. The return rate of probes and questionnaires was complete for both groups (preterm *n* = 25; controls *n* = 24) at all timepoints. All probes were included in the statistical analyses.

**FIGURE 2:**
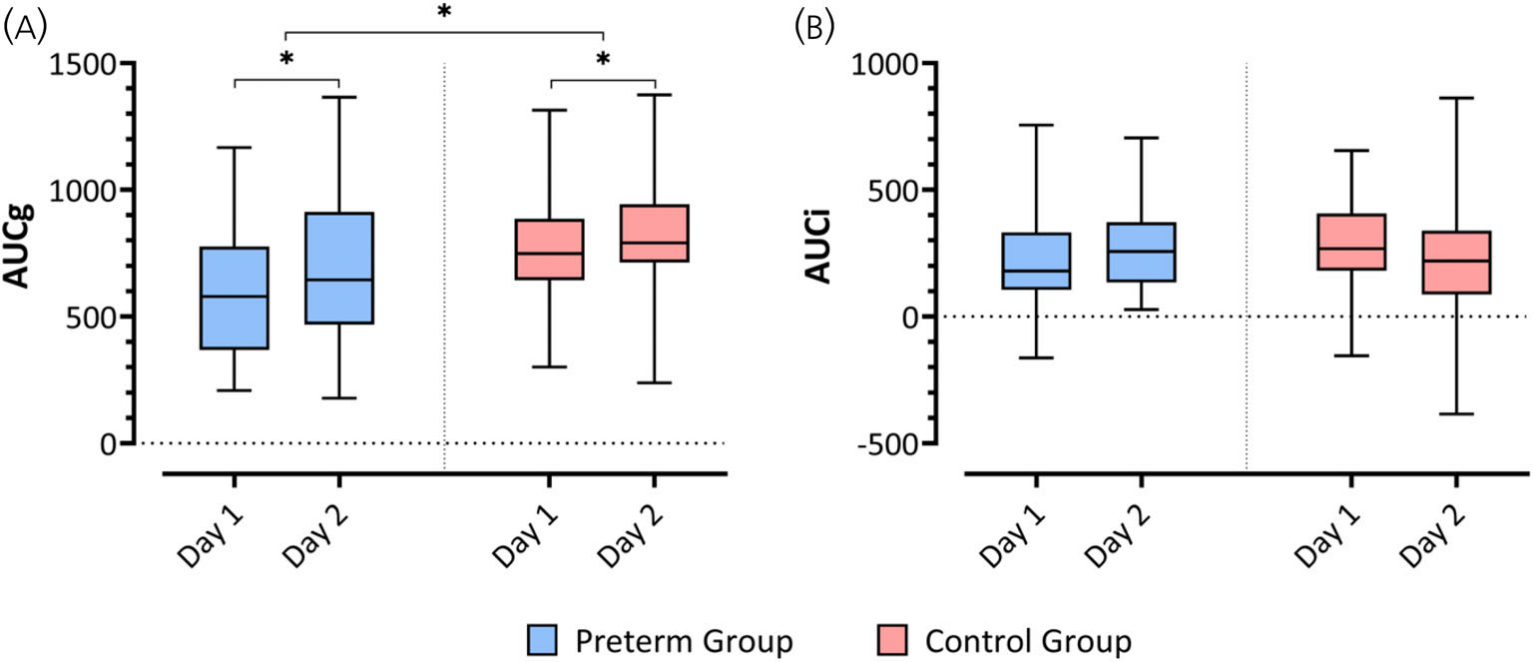
Box plots presenting results of (A) AUCg and (B) AUCi results on Day 1 and Day 2 of the preterm (blue) and control group (red). (A) AUCg values were significantly higher in control compared to the preterm group (*p* = .04, least squares means test) and significantly higher on Day 2 compared to Day 1 (*p* = .02, least squares means test) for both groups. (B) Post hoc analysis revealed higher AUCi values on Day 1 compared to Day 2 (*p* = .047, least squares means test); however, this significance did not survive the correction for multiple comparisons (*p* = .28, least squares means test). AUCg, area under the curve with respect to the ground; AUCi, area under the curve with respect to the increase.

The RMANOVA yielded a significant effect of group on AUCg (*F*_1,47_ = 4.51, *p* = .04) and Day (*F*_1,47_ = 6.07, *p* = .017), but no significant interaction (*F*_1,47_ = 1.30, *p* = .26). AUCg values were significantly higher in the control compared to the preterm group (*t*_47_ = −2.85; *p* = .04) and significantly higher on Day 2 compared to Day 1 (*t*_47_ = −2.46; *p* = .02, least squares means test) in both groups (Figure 2). Regarding AUCi, the RMANOVA revealed a significant Group × Day interaction (*F*_1,47_ = 6.58, *p* = .01), but no significant main effect of Group (*F*_1,47_ = 0.12, *p* = .73) or Day (*F*_1,47_ = 0.30, *p* = .59). Post hoc analyses revealed a higher AUCi on Day 1 compared to Day 2 (*t*_47_ = 2.04, *p* = .047, least squares means test). However, this finding did not remain significant after correction for multiple comparisons (*p* = .28, least squares means test) (Figure 2). Table S3 shows the mean values and SD for AUCg, AUCi for the preterm and control group.

On Day 1, absolute mean saliva cortisol concentration rose from 8.24 to 15.35 nmol/L in controls (+86.69%) and from 5.66 to 10.85 nmol/L in preterms (+91.7%). On Day 2, cortisol concentration rose from 10.4 to 16.67 nmol/L in controls (+60.29%) and from 6.71 to 13.62 nmol/L in preterms (+102.98%).

The RMANOVA on absolute saliva cortisol concentration conducted on each day separately revealed significant effects of Group (Day1: *F*_1,188_ = 18.09, *p* < .001, Day2: *F*_1,188_ = 4.68, *p* = .03) and Time (Day1: *F*_3,188_ = 19.46, *p* < .001, Day2: *F*_3,188_ = 14.15, *p* < .001), but not of the Group × Time interaction (Day1: *F*_3,188_ = 0.36, *p* = .78; Day2: *F*_3,188_ = 0.99, *p* = .398). On both days, values of S1 were significantly lower in preterm born adults (Day1: *t*_188_ = −4.25, *p* < .01; Day2: *t*_188_ = −2.16, *p* = .032, least squares means test). On Day 1 S1 values were significantly lower at *t* = 0 min compared to S1 values at *t* = 30 (*t*_188_ = −4.84, *p* < .001, least squares means test), *t* = 45 (*t*_188_ = −6.35, *p* < .001, least squares means test), and *t* = 60 min (*t*_188_ = −4.06, *p* < .001, least squares means test), as well as at *t* = 60 min compared to *t* = 30 min (*t*_188_ = 2.78, *p* = .036, least squares means test). On Day 2 S1 values were significantly lower at *t* = 0 min compared to S1 values at *t* = 30 (*t*_188_ = −5.90, *p* < .001, least squares means test) and *t* = 45 min (*t*_188_ = −4.66, *p* < .001, least squares means test), as well as at *t* = 60 min compared to *t* = 30 (*t*_188_ = −3.97, *p* < .001, least squares means test) and *t* = 45 min (*t*_188_ = −2.72, *p* = .043, least squares means test).

Saliva cortisol level at *t* = 0 min significantly correlated with birth weight (Day 1: *p* = .01, *r* = 0.36; Day 2 *p* = .012, *r* = 0.34) and with gestational age (Day 1: *p* = .02, *r* = 0.34; Day 2: *p* = .03, *r* = 0.32).

### 3.3 Differences in State–trait‐anxiety and perceived stress between preterm born adults and controls

**FIGURE 3:**
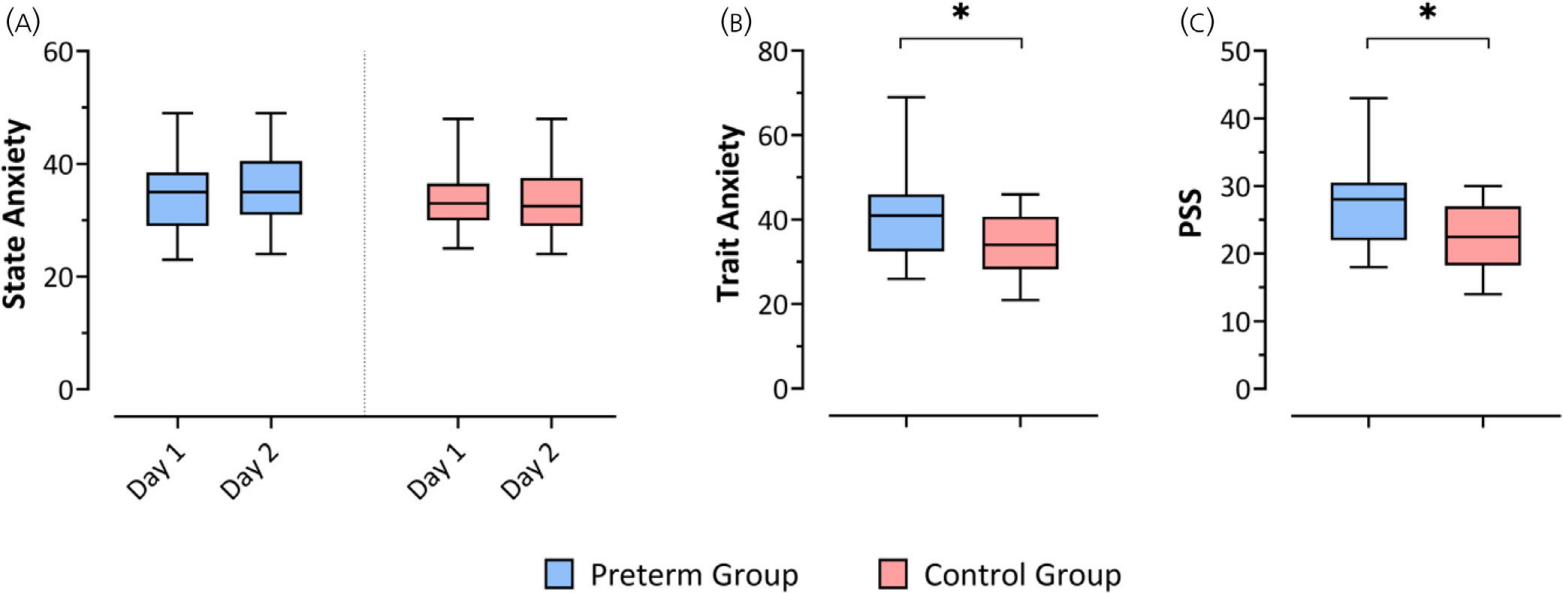
Box plots presenting results of the STAI and the PSS in the preterm (blue) and term group (red). (A) State Anxiety on Day 1 and 2 with no significant differences between the preterm and control group. (B) Trait anxiety was significantly higher in the preterm group (*U* = 175.5; *p* = .01; Mann–Whitney *U* test). (C) PSS shows significant higher levels in the preterm group (*U* = 172.5; *p* = 0.01; Mann–Whitney *U* test). Data are presented as median and range. *n* = 25 for the preterm and *n* = 24 for controls. PSS, perceived stress scale.

There were no significant differences between the preterm and control group in terms of state anxiety (Day 1: *U* = 293.5; *p* = .91; Day 2: *U* = 216; *p* = .1). Furthermore, there were no significant differences when comparing state anxiety results of Day 1 and Day 2. Trait anxiety was significantly higher for the preterm group (*U* = 175.5; *p* = .01; Mann–Whitney *U* test). PSS was significantly higher in the preterm group (*U* = 172.5; *p* = .01; Mann–Whitney *U* test) (Figure 3).

### 3.4 Correlations between the CAR and STAI as well as PSS

There was a significant positive correlation between state anxiety and AUCg for preterm (*r* = 0.39954, *p* = .0478), but not for controls (*r* = −0.023922, *p* = .26) on Day 1. There were no further significant correlations in the preterm group.

In the control group, there was a significant negative correlation (*r* = −0.47, *p* = .02) between AUCg and trait anxiety on Day 2. On the same day, a significant positive correlation between PSS and AUCi was observed in the control group (*r* = 0.64, *p* = .001) as well as a negative correlation between S1 and trait anxiety (*r* = −0.66, *p* = .0005) as well as PSS (*r* = −0.56, *p* = .0046) results. Please see Table 2 for the additional information on the correlation statistics.

**TABLE 2:** Correlation statistics and slope coefficients for the AUCg, AUCI, and S1 (first cortisol level upon awakening at 0 min).

Measure pair	Group	Day	Slope coefficient	*r*	*R* ^2^	*p*	Adj *p*
AUCg/state anxiety	Preterm	Day 1	17.10	**0.4**	0.16	.**048**	.14
		Day 2	2.39	0.05	0.003	.81	1.00
	Control	Day 1	−9.21	−0.21	0.05	.32	.95
		Day 2	−11.05	−0.24	0.06	.26	.78
AUCi/state anxiety	Preterm	Day 1	7.41	0.23	0.05	.27	.82
		Day 2	1.65	0.06	0.003	.8	1.0000
	Control	Day 1	4.14	0.12	0.01	.58	1.0000
		Day 2	7.52	0.17	0.03	.44	1.0000
AUCg/trait anxiety	Preterm	Day 1	−0.83	−0.03	0.001	.88	1.0000
		Day 2	−6.59	−0.22	0.05	.28	.85
	Control	Day 1	−10.30	−0.35	0.12	.09	.281
		Day 2	−16.55	**−0.47**	0.2	.**02**	.06
AUCi/trait anxiety	Preterm	Day 1	−1.82	−0.095	0.01	.65	1.0000
		Day 2	−0.95	−0.05	0.003	.81	1.0000
	Control	Day 1	−0.06	−0.002	<0.0001	.99	1.0000
		Day 2	12.20	0.35	0.13	.09	.27
AUCg/Perceived Stress Scale	Preterm	Day 1	3.42	0.08	0.01	.70	1.00
		Day 2	−10.01	−0.20	0.04	.33	.996
	Control	Day 1	−2.88	−0.07	0.005	.76	1.00
		Day 2	−3.78	−0.07	0.005	.73	1.00
AUCi/Perceived Stress Scale	Preterm	Day 1	1.10	0.03	0.001	.87	1.00
		Day 2	−2.67	−0.09	0.007	.69	1.00
	Control	Day 1	6.36	0.18	0.03	.4	1.00
		Day 2	32.05	**0.64**	0.41	.**0008**	.002
S1/state anxiety	Preterm	Day 1	0.16	0.34	0.11	.09	.28
		Day 2	0.01	0.01	0.0004	.92	1.00
	Control	Day 1	−0.22	0.28	0.08	.18	.54
		Day 2	−0.31	−0.32	0.10	.13	.38
S1/trait anxiety	Preterm	Day 1	0.02	0.06	0.004	.78	1.00
		Day 2	−0.09	−0.25	0.06	.22	.67
	Control	Day 1	−0.17	−0.32	0.10	.13	.38
		Day 2	−0.48	**−0.66**	0.43	.**0005**	.0015
S1/Perceived Stress Scale	Preterm	Day 1	0.039	0.08	0.007	.69	1.00
		Day 2	−0.12	−0.2	0.038	.35	1.00
	Control	Day 1	0.16	−0.2	0.039	.36	1.00
		Day 2	−0.6	**−0.56**	0.31	.**0046**	.014

*Note*: Bold indicates significant value (*p* < .05).

## 4 DISCUSSION

The primary aim of this study was to investigate possible differences of the CAR between very preterm born adults and term born adults. The results do not suggest differences in the actual CAR represented by the AUCi. However, the preterm group exhibited significantly lower cortisol levels at awakening (S1, 0 min). Furthermore, the total cortisol output, represented by the AUCg during the first morning hour, was significantly lower in the preterm group.

Few studies have assessed the CAR in former preterm born infants, and existing results regarding long‐term alterations remain inconclusive as outlined below. In line with our results, Maurer et al. found significantly lower salivary cortisol levels at awakening (S1) and lower overall post‐awakening cortisol secretion (AUCg) in a cohort of 85 infants born very preterm (<32nd gestational week) at the age of 7–12 years, compared to term born children, without differences in the AUCi, indicating a comparable CAR. The preterm group also demonstrated lower cortisone in hair.^16^ A recent study by Watterberg et al.^17^ found blunted morning cortisol levels at awakening and 30 min later in a large cohort (*n* = 219) of extremely preterm (24–27 weeks GA) infants at the age of 6–7 years, compared to term‐born controls, though AUCg or AUCi values were not reported.^17^ These lower cortisol levels were associated with memory and inattention problems.^25^ It is hypothesized that lower cortisol values in former very preterm infants are caused by a downregulation of the HPA axis activity as consequence of prolonged postnatal stress.^16^, ^26^ In a study by Brummelte et al., lower cortisol levels in school aged children were associated with higher neonatal stress and pain caused by skin‐breaking procedures.^27^

In contrast to these findings, Quesada et al. found significantly higher total salivary cortisol concentrations immediately after awakening and a lower CAR in former preterm infants of 26–36 weeks' GA at the age of 6–10 years compared to age‐ and sex‐matched controls.^28^ However, the cohort was relatively small (31 preterm born children), and a subgroup analysis indicated that higher morning cortisol levels were more pronounced among preterm‐born girls, likely driving this finding.^28^ In our relatively small cohort, no significant sex effect on saliva cortisol levels could be measured (Table S1). Additionally, Buske‐Kirschbaum et al. assessed the CAR in former preterm infants at the age of 8–14 years in comparison with sex‐ and age‐matched full‐term born controls. The study also showed significantly higher cortisol levels after awakening but did not report AUCi data. In this cohort, gestational age ranged between 26 and 36 weeks and 16 (89%) were small for gestational age (SGA).^15^ The higher gestational ages and a higher proportion of SGA may explain these differences, compared to the present study, as particularly intra‐uterine growth restriction, including SGA status, has been linked to increased basal cortisol levels and increased cortisol responses to stress.^29^, ^30^ In the present study, only one preterm participant was born SGA.

Beyond childhood, Gustafsson et al. investigated the relation between birth weight and circadian salivary cortisol levels in adulthood.^31^ The study results indicated a positive relation between birth weight and total cortisol as well as bedtime levels in a cohort of 43 years old adults born in 1965. While preterm infants or infants with birth weight <2500 g were excluded from main analyses, an explorative analysis of this subgroup showed elevated evening and total cortisol levels comparable with infants >3999 g. Relating these findings to our study results is difficult because the time points of saliva collection were different (at awakening, 15 min after awakening, before lunch and before bedtime). While our study only included very preterm infants (born <32 weeks' gestation, mean birth weight 1240 g), there is no further information on the preterm group analyzed by Gustaffson et al. (except of birth weight <2500 g, which could also include small for gestational age born at term). Furthermore, the preterm group was born in 1965 and neonatal care has significantly changed, especially since the introduction of surfactant in the 1990s, which led to decreased mortality and a shift of the border of viability to more immature infants in high‐income countries.^32^

Since the CAR has been linked to a variety of psychosocial, physical and mental health problems,^19^ our secondary aim was to investigate possible associations between the morning cortisol profile and anxiety and stress. We found higher trait anxiety and perceived stress levels in the preterm group, thus confirming our hypothesis. In the preterm group, we found a significant positive correlation between the AUCg and state anxiety on Day 1. In the control group, a significant negative correlation were noted on Day 2 between AUCg and trait anxiety, a positive correlation between AUCi and perceived stress scale (PSS) scores, and a negative correlation between S1 and both trait anxiety and PSS scores. These inconsistent correlations between Days 1 and 2 may be explained by the relatively small cohort size. Furthermore, not only trait but also state related factors, in particular psychosocial variables, can influence morning cortisol levels and the CAR.^19^ In the current study, we only assessed state anxiety and sleepiness as possible state factors, neither of which were significantly different between Days 1 and 2.

## 5 LIMITATIONS

The sample size was small as it relied on the primary study on fear conditioning and only 25/38 preterm‐born adults and 24/42 term‐born adults agreed to participate in this extension^18^ study. Thus, larger studies are necessary to strengthen evidence regarding altered cortisol morning profile in very preterm‐born young adults. A further limitation is the domestic collection of salivary cortisol, which might have affected data reliability, despite ecological validity. However, following the expert consensus guidelines on the assessment of the CAR,^19^ we tried to maximize adherence through comprehensive written and video instructions, along with a checklist completed during sampling.

## CONCLUSION

The study results suggest long term alterations of the HPA axis with decreased total morning salivary cortisol levels but normal CAR in very preterm born adults. These results are in line with studies in very preterm born children. The inconsistent results on the associations between the CAR and stress or anxiety in this study, do not allow to draw a definitive conclusion. Larger studies are needed to confirm these results, further investigate the unclear issues and to clarify its clinical relevance in this population.

## AUTHOR CONTRIBUTIONS

**Bilge Albayrak:** Conceptualization; investigation; funding acquisition; writing – original draft; writing – review and editing; supervision; resources; data curation. **Giorgi Batsikadze:** Methodology; formal analysis; software; data curation. **Lara Jablonski:** Project administration. **Ursula Felderhoff‐Müser:** Writing – review and editing; supervision. **Tina Hörbelt‐Grünheidt:** Conceptualization; methodology. **Anna Lena Friedel:** Methodology; conceptualization. **Raphael Hirtz:** Methodology; conceptualization. **Katharina Heuser‐Spura:** Conceptualization; formal analysis; methodology; validation; data curation. **Monia V. Dewan:** Conceptualization; funding acquisition; writing – original draft; writing – review and editing; supervision; visualization; data curation.

## FUNDING INFORMATION

This study was supported by the UMEA (University Medicine Essen Clinician Scientist Academy) FU 356/12‐2.

## CONFLICT OF INTEREST STATEMENT

The authors declare no conflicts of interest.

### PEER REVIEW

The peer review history for this article is available at https://www.webofscience.com/api/gateway/wos/peer-review/10.1111/jne.70000.

## Supporting information


Table S1.

Table S2.

Table S3.

## Data Availability

The data that support the findings of this study are available on request from the corresponding author. The data are not publicly available due to privacy or ethical restrictions.

## References

[1] Dobbing J , Sands J . Comparative aspects of the brain growth spurt. Early Hum Dev. 1979;3:79‐83. doi:10.1016/0378-3782(79)90022-7 118862

[2] Wood NS , Marlow N , Costeloe K , Gibson AT , Wilkinson AR . Neurologic and developmental disability after extremely preterm birth. N Engl J Med. 2000;343:378‐384. doi:10.1056/NEJM200008103430601 10933736

[3] Johnson S , Marlow N . Preterm birth and childhood psychiatric disorders. Pediatr Res. 2011;69:11‐18. doi:10.1203/PDR.0b013e318212faa0 21289534

[4] Mathewson KJ , Chow CHT , Dobson KG , Pope EI , Schmidt LA , Van Lieshout RJ . Mental health of extremely low birth weight survivors: a systematic review and meta‐analysis. Psychol Bull. 2017;143:347‐383. doi:10.1037/bul0000091 28191983

[5] Benestad MR , Drageset J , Hufthammer KO , Vollsæter M , Halvorsen T , Vederhus BJ . Long‐term follow‐up of self‐reported mental health and health‐related quality of life in adults born extremely preterm. Early Hum Dev. 2022;173:105661. doi:10.1016/j.earlhumdev.2022.105661 36067714

[6] Faravelli C , Lo Sauro C , Godini L , et al. Childhood stressful events, HPA axis and anxiety disorders. World J Psychiatry. 2012;2:13‐25. doi:10.5498/wjp.v2.i1.13 24175164 PMC3782172

[7] Finken MJJ , van der Voorn B , Hollanders JJ , et al. Programming of the hypothalamus–pituitary–adrenal axis by very preterm birth. Ann Nutr Metab. 2017;70:170‐174. doi:10.1159/000456040 28301846 PMC5516415

[8] Ng PC . Adrenocortical insufficiency and refractory hypotension in preterm infants. Arch Dis Child Fetal Neonatal ed. 2016;101:F571‐F576. doi:10.1136/archdischild-2016-311289 27601464

[9] Stoye DQ , Boardman JP , Osmond C , et al. Saliva cortisol diurnal variation and stress responses in term and preterm infants. Arch Dis Child Fetal Neonatal Ed. 2022;107:558‐564. doi:10.1136/archdischild-2021-321593 35256524 PMC9411886

[10] Urfer A , Turpin H , Dimitrova N , et al. Consequences of prematurity on cortisol regulation and adjustment difficulties: a 9‐year longitudinal study. Children. 2021;9(1):9. doi:10.3390/children9010009 35053633 PMC8774148

[11] Lammertink F , Vinkers CH , Tataranno ML , Benders MJNL . Premature birth and developmental programming: mechanisms of resilience and vulnerability. Front Psychiatry. 2021;11:531571. doi:10.3389/fpsyt.2020.531571 33488409 PMC7820177

[12] Vinall J , Grunau RE . Impact of repeated procedural pain‐related stress in infants born very preterm. Pediatr Res. 2014;75:584‐587. doi:10.1038/pr.2014.16 24500615 PMC3992189

[13] Fries E , Dettenborn L , Kirschbaum C . The cortisol awakening response (CAR): facts and future directions. Int J Psychophysiol Off J Int Organ Psychophysiol. 2009;72:67‐73. doi:10.1016/j.ijpsycho.2008.03.014 18854200

[14] Gao H , Liu X , Gou L , Jing J , Qi M . High trait anxiety predicts decreased cortisol awakening response. J Psychopathol Behav Assess. 2024;46:252‐259. doi:10.1007/s10862-023-10045-9

[15] Buske‐Kirschbaum A , Krieger S , Wilkes C , Rauh W , Weiss S , Hellhammer DH . Hypothalamic–pituitary–adrenal axis function and the cellular immune response in former preterm children. J Clin Endocrinol Metab. 2007;92:3429‐3435. doi:10.1210/jc.2006-2223 17566098

[16] Maurer N , Perkinson‐Gloor N , Stalder T , et al. Salivary and hair glucocorticoids and sleep in very preterm children during school age. Psychoneuroendocrinology. 2016;72:166‐174. doi:10.1016/j.psyneuen.2016.07.003 27434634

[17] Watterberg KL , Hintz SR , Do B , et al. Adrenal function links to early postnatal growth and blood pressure at age 6 in children born extremely preterm. Pediatr Res. 2019;86(3):339‐347. doi:10.1038/s41390-018-0243-1 30631138 PMC6561840

[18] Albayrak B , Jablonski L , Felderhoff‐Mueser U , et al. Fear conditioning is preserved in very preterm‐born young adults despite increased anxiety levels. Sci Rep. 2023;13:11319. doi:10.1038/s41598-023-38391-4 37443342 PMC10344879

[19] Stalder T , Kirschbaum C , Kudielka BM , et al. Assessment of the cortisol awakening response: expert consensus guidelines. Psychoneuroendocrinology. 2016;63:414‐432. doi:10.1016/j.psyneuen.2015.10.010 26563991

[20] Weigl T , Schneider N , Stein A , Felderhoff‐Müser U , Schedlowski M , Engler H . Postpartal affective and endocrine differences between parents of preterm and full‐term infants. Front Psychiatry. 2020;11:251. doi:10.3389/fpsyt.2020.00251 32296356 PMC7139630

[21] Spielberger CD . State–Trait Anxiety Inventory for Adults; 2012. APA PsycTests. doi:10.1037/t06496-000

[22] Schneider EE , Schönfelder S , Domke‐Wolf M , Wessa M . Measuring stress in clinical and nonclinical subjects using a German adaptation of the perceived stress scale. Int J Clin Health Psychol. 2020;20(2):173‐181. doi:10.1016/j.ijchp.2020.03.004 32550857 PMC7296237

[23] Shahid A , Wilkinson K , Marcu S , Shapiro CM . Stanford Sleepiness Scale (SSS). In: Shahid A , Wilkinson K , Marcu S , Shapiro C , eds. STOP, THAT and One Hundred Other Sleep Scales. Springer; 2011. doi:10.1007/978-1-4419-9893-4_91

[24] Nasser A , Ozenne B , Høgsted ES , Jensen PS , Frokjaer VG . Reliability of three versus five saliva sampling times for assessing the cortisol awakening response. Psychoneuroendocrinology. 2023;147:105950. doi:10.1016/j.psyneuen.2022.105950 36272363

[25] Lowe J , Fuller JF , Dempsey AG , et al. Cortisol awakening response and developmental outcomes at 6–7 years in children born extremely preterm. Pediatr Res. 2023;93:689‐695. doi:10.1038/s41390-022-02113-9 35715492 PMC9758271

[26] Wadsby M , Nelson N , Ingemansson F , Samuelsson S , Leijon I . Behaviour problems and cortisol levels in very‐low‐birth‐weight children. Nord J Psychiatry. 2014;68:626‐632. doi:10.3109/08039488.2014.907341 24802123

[27] Brummelte S , Chau CMY , Cepeda IL , et al. Cortisol levels in former preterm children at school age are predicted by neonatal procedural pain‐related stress. Psychoneuroendocrinology. 2015;51:151‐163. doi:10.1016/j.psyneuen.2014.09.018 25313535 PMC4268136

[28] Quesada AA , Tristão RM , Pratesi R , Wolf OT . Hyper‐responsiveness to acute stress, emotional problems and poorer memory in former preterm children. Stress. 2014;17:389‐399. doi:10.3109/10253890.2014.949667 25089937

[29] Cianfarani S , Geremia C , Scott CD , Germani D . Growth, IGF system, and cortisol in children with intrauterine growth retardation: is catch‐up growth affected by reprogramming of the hypothalamic–pituitary–adrenal Axis? Pediatr Res. 2002;51:94‐99. doi:10.1203/00006450-200201000-00017 11756646

[30] Iwata S , Kinoshita M , Okamura H , et al. Intrauterine growth and the maturation process of adrenal function. PeerJ. 2019;7:e6368. doi:10.7717/peerj.6368 30746307 PMC6368969

[31] Gustafsson PE , Janlert U , Theorell T , Hammarström A . Is body size at birth related to circadian salivary cortisol levels in adulthood? Results from a longitudinal cohort study. BMC Public Health. 2010;10:346. doi:10.1186/1471-2458-10-346 20553630 PMC2908578

[32] Hallman M , Herting E . Historical perspective on surfactant therapy: transforming hyaline membrane disease to respiratory distress syndrome. Semin Fetal Neonatal Med. 2023;28(6):101493. doi:10.1016/j.siny.2023.101493 38030434

