# Supplementary material for: The cortisol awakening response in very preterm born adults compared to term born adults
Source: J Neuroendocrinol. 2025 Feb 10;37(4):e70000. doi: 10.1111/jne.70000 (PMC11975800; doi:10.1111/jne.70000)
Supplement: Supplementary file 1 — Table S1. [file JNE-37-e70000-s002.docx]

Supplemental Table S1. RM-ANOVA for AUCg/AUCi and Sex / Contraceptive use as a covariates

| **Factor** | **Num Df** | | **Den Df** | | | **F** | | **P** |
| --- | --- | --- | --- | --- | --- | --- | --- | --- |
| **AUCg** | | | | | | | | |
| Group  Day  Group ´ Day  Sex | | 1  1  1  1 | | 47  47  47  47 | 4.38  6.07  1.30  0.21 | | **0.042***  **0.017***  0.260  0.652 | |
| Group  Day  Group ´ Day  Contraceptive Use | | 1  1  1  1 | | 47  47  47  47 | 4.12  6.07  1.30  0.49 | | **0.048***  **0.017***  0.260  0.487 | |
| **AUCi** | | | | | | | | |
| Group  Day  Group ´ Day  Sex | | 1  1  1  1 | | 47  47  47  47 | 0.14  0.30  6.60  1.50 | | 0.707  0.588  **0.013***  0.226 | |
| Group  Day  Group ´ Day  Contraceptive Use | | 1  1  1  1 | | 47  47  47  47 | 0.23  0.30  6.62  1.77 | | 0.631  0.588  **0.013***  0.190 | |
| ***** Significant results at *p* < 0.05. | | | | | | | | |
|  |  |  |  |  |  |  |  |  |
