# Supplementary material for: The cortisol awakening response in very preterm born adults compared to term born adults
Source: J Neuroendocrinol. 2025 Feb 10;37(4):e70000. doi: 10.1111/jne.70000 (PMC11975800; doi:10.1111/jne.70000)
Supplement: Supplementary file 2 — Table S2. [file JNE-37-e70000-s003.docx]

| Supplemental Table S2. *Group characteristics of very preterm adults and controls comparing initial and final cohort* | | | | | | | | |
| --- | --- | --- | --- | --- | --- | --- | --- | --- |
|  | Very preterm | | | *p*^a^ | Term | | | *p*^a^ |
|  | Initial cohort  (n = 37) | Final cohort  (n = 25) | Excluded cohort  (n=12) |  | Initial cohort  (n = 31) | Final cohort  (n = 24) | Excluded cohort  (n=7) |  |
| Clinical characteristics | | | | | | | | |
| Gestational age, weeks [range] | 29+2.8  [26+2 – 32.0] | 29+1  [26+2–32.0] | 28+6  [26+2–32.0] | 0.2 | 39+5.7  [37.0 – 42.0] | 39+5.7  [37.0 – 42.0] | 39+6  [38.0+4 – 41.0] | 0.35 |
| Birth weight, grams [range] | 1264.2  [520 – 2370] | 1240.4  [850 – 1750] | 1149  [520 - 1680] | 0.33 | 3637.5  [2995 – 5360] | 3648.3  [3020 – 5360] | 3640  [3060 – 4290] | 0.47 |
| Female, *n* (%) | 19 (51.4%) | 14 (56%) | 5 (41.7%) | 0.5 | 16 (51.6%) | 14 (58.3%) | 2 (28.6%) | 0.22 |
| IVH < grad III, *n* (%) | 1 (2.7%) | 1 (4%) | 0 (0%) | 0.78 | 0 (0%) | 0 (0%) | 0 (0%) | 1.0 |
| Follow – up characteristics | | | | | | | | |
| Age at assessment, years [range] | 20.2  [17.8 – 27] | 20.5  [17.8 – 27] | 19.1  [17.8-22] | 0.06 | 22.4  [18 – 29] | 22.3  [18 – 25] | 22  [18 – 25] | 0.3 |
| Education, high^b^*, n* | 30 | 19 | 11 | 0.39 | 29 | 24 | 5 | **0.05** |
| Parental education, high^b^, *n* | 22 | 22 | 0 | **<0.01** | 24 | 24 | 0 | **<0.01** |
| Health status^c^ | 5.88  [4.14 – 7] | 5.61  [3.71 – 6.57] | 5.06  [4.22-6.37] | 0.09 | 5.47  [3.71 – 6.57] | 5.82  [4.14 – 7] | 5.32  [4.46 – 6.24] | 0.07 |
| IQ | 97  [ 83 – 123] | 100.4  [88 – 123] | 101.2  [83 - 123] | 0.36 | 108.71  [89 – 122] | 108.5  [89 – 122] | 108.9  [101 – 115] | 0.47 |
| Any therapy^d^, *n* (%) | 15 (40.5%) | 15 (60%) | 0 | <**0.01** | 0 (0%) | 0 (0%) | 0 | 1.0 |
| Any psychiatric/ social – emotional disorders^e^, *n* (%)   - Phobias/ anxiety/ depression, n (%) - ADS/ ADHS, n (%) | 12 (32.4%)  5 (13.5%)  5 (13.5%) | 6 (24%)  1 (4%)  5 (20%) | 6 (50%)  4 (33.3%)  0 (0%) | 0.15  **0.03**  0.15 | 1 (3.2%)  0 (0%)  0 (0%) | 1 (4.2%)  0 (0%)  0 (0%) | 0 (0%)  0 (0%)  0 (0%) | 1.0  1.0  1.0 |
| Developmental disorders^f^, *n* (%) | 15 (40.5%) | 4 (16%) | 11 (91.7%) | **<0.01** | 0 (0%) | 0 (0%) | 0 (0%) | 1.0 |
| BMI | 21.8  [17.9 – 27.8] | 21.9  [18 – 28.4] | 20.72  [17.9 – 27.8] | 0.08 | 23  [18.8 – 30.8] | 22.6  [18.8 – 27.5] | 21.42  [19 – 24.5] | **0.02** |
| Use of contraceptives | 12 (63.2 %) | 11 (78.6%) | 1 (8.3%) | **0.04** | 14 (87.5%) | 13 (92.3%) | 1 (50%) | 0.24 |
| *Notes*. Data are presented as mean (standard deviation) if not indicated otherwise.  *^a^ t – test or Mann – Whitney U – test and chi – square results or Fischer’s exact test for continuous and categorical data, respectively.*  ^b^ > 10 years school.  ^c^ assessment of health status based on the *Life Satisfaction Questionnaire (Fragebogen zur Lebenszufriedenheit, FLZ)*  ^d^ having any therapies, including speech therapy, physical therapy, or occupational therapy.  ^e^ psychiatric disorders including attention – deficit – (hyperactivity) – disorder, emotional disorder.  SGA – Small for gestational age (birth weight <10^th^ percentile), IVH – Intraventricular haemorrhage, BPD – Bronchopulmonary dysplasia, ROP – Retinopathy of prematurity, IQ – Intelligence quotient based on the *Wechsler Adult Intelligence Scale – Third Edition*  ^f^ developmental disorders concerning language, gross – or fine motor functions which needed therapy in the past (speech therapy, physical therapy or occupational therapy) | | | | | | | | |
