# Supplementary material for: The cortisol awakening response in very preterm born adults compared to term born adults
Source: J Neuroendocrinol. 2025 Feb 10;37(4):e70000. doi: 10.1111/jne.70000 (PMC11975800; doi:10.1111/jne.70000)
Supplement: Supplementary file 3 — Table S3. [file JNE-37-e70000-s001.docx]

Supplemental Table S3.

| **Measure** | **Preterm group** | | **Control group** | |
| --- | --- | --- | --- | --- |
|  | **Day 1** | **Day 2** | **Day 1** | **Day 2** |
| AUCg (mean ± SD) | 590.29 ± 252.95 | 682.32 ± 297.08 | 765.23 ± 215.33 | 799.01 ± 256.96 |
| AUCi (mean ± SD) | 228.03 ± 191.91 | 282.04 ± 189.24 | 278.59 ± 174.95 | 195.47 ± 251.73 |

mean values and SD for AUCg, AUCi for the preterm and control group.
